# A Case of Heart Clot

**Published:** 1880-04

**Authors:** 


### EXCERPTA.

A CASE OF HEART CLOT.—N. B, aged fifty-seven, a large, very adipose man, who had been accustomed until six years ago to perform hard manual labor. He was disabled six years ago by a very severe injury of the head, which also produced an immense inguinal hernia that rendered walking very difficult.

The patient had been under treatment for some time for chronic rheumatism, and on September 12 I was called to see him. His rheumatic symptoms had about disappeared, but on the day preceding the one on which I was called he had an attack of quite severe headache, which still continued, though somewhat abated. At my next visit, three days later, I learned that the headache had disappeared, but he seemed dull, disinclined to move or speak, and did not rest well at night; pulse was 104, and temperature 100°. From the third to the eighth day the symptoms gradually became more severe, his temperature ranging from 100 to 102½°, pulse from 90 to 120, tongue slightly coated, bowels constipated, except as moved by cathartics, restless at night.

On the seventh day slight cyanosis of the face was observed, On the eighth, the diagnosis of passive cerebral congestion from obstruction in the heart was made. Council was called, and a thorough examination of the lungs and heart was made, but gave a negative result. Council did not concur in the diagnosis.

On the tenth day the symptoms were well marked, and were as follows: A peculiar attitude, the head resting on the chest when sitting up; face cyanotic; eyes staring; a perfectly blank expression; coated tongue; rapid pulse; marked stupor, so that he would not utter more than one word before relapsing into his apathetic condition from which he could not be again aroused without considerable effort; articulation indistinct and thick; respiration peculiar—a half dozen respirations being very superficial, scarcely perceptible, followed by as many very deep and noisy, as though the respiratory centers did not respond to the stimulus of thc ordinary amount of carbonic acid; the length of the intervals between the succession of noisy respirations constantly increasing.

Death took place on the thirteenth day from apnœa.

Postmortem examination revealed a fibrinous clot in the right auricle of a pale pink color, quite firm under pressure, about one and a half inches in length, three-fourths inches in breadth, and about a fourth of an inch in thickness; also a soft, dark coagulum, about two inches in length, in the pulmonary artery. Evidently this was of postmortem formation, but the extremity nearest the heart was firmer, and of a pink color, similar to the one in the right uricle.

No other lesions were discovered.

